# The effect of corticosteroids on mortality of patients with influenza pneumonia: a systematic review and meta-analysis

**DOI:** 10.1186/s13054-019-2395-8

**Published:** 2019-03-27

**Authors:** Yue-Nan Ni, Guo Chen, Jiankui Sun, Bin-Miao Liang, Zong-An Liang

**Affiliations:** 1 Department of Respiratory and Critical Care Medicine, West China School of Medicine and West China Hospital, Sichuan University, No. 37 Guoxue Alley, Chengdu, 610041 Sichuan China; 2 Department of Geriatrics, Sichuan Academy of Medical Sciences & Sichuan Provincial People’s Hospital, Chengdu, Sichuan China; 3 State Key Laboratory of Oral Diseases, West China School of Stomatology, Sichuan University, No. 14, Section 3 Renmin Nanlu, Chengdu, 610041 Sichuan China

**Keywords:** Corticosteroids, Influenza pneumonia, Mortality

## Abstract

**Background:**

The effect of corticosteroids on clinical outcomes in patients with influenza pneumonia remains controversial. We aimed to further evaluate the influence of corticosteroids on mortality in adult patients with influenza pneumonia by comparing corticosteroid-treated and placebo-treated patients.

**Methods:**

The PubMed, Embase, Medline, Cochrane Central Register of Controlled Trials (CENTRAL), and Information Sciences Institute (ISI) Web of Science databases were searched for all controlled studies that compared the effects of corticosteroids and placebo in adult patients with influenza pneumonia. The primary outcome was mortality, and the secondary outcomes were mechanical ventilation (MV) days, length of stay in the intensive care unit (ICU LOS), and the rate of secondary infection.

**Results:**

Ten trials involving 6548 patients were pooled in our final analysis. Significant heterogeneity was found in all outcome measures except for ICU LOS (*I*^2^ = 38%, *P* = 0.21). Compared with placebo, corticosteroids were associated with higher mortality (risk ratio [RR] 1.75, 95% confidence interval [CI] 1.30 ~ 2.36, *Z* = 3.71, *P* = 0.0002), longer ICU LOS (mean difference [MD] 2.14, 95% CI 1.17 ~ 3.10, *Z* = 4.35, *P* < 0.0001), and a higher rate of secondary infection (RR 1.98, 95% CI 1.04 ~ 3.78, Z = 2.08, *P* = 0.04) but not MV days (MD 0.81, 95% CI − 1.23 ~ 2.84, *Z* = 0.78, *P* = 0.44) in patients with influenza pneumonia.

**Conclusions:**

In patients with influenza pneumonia, corticosteroid use is associated with higher mortality.

**Trial registration:**

PROSPERO (ID: CRD42018112384).

**Electronic supplementary material:**

The online version of this article (10.1186/s13054-019-2395-8) contains supplementary material, which is available to authorized users.

## Introduction

Influenza virus infections cause excessive hospitalizations and deaths among adults during seasonal peaks and pandemics. Among all patients infected with H7N9, 97% presented with rapidly progressive pneumonia, and 71% presented with pneumonia caused by influenza virus infection and complicated by acute respiratory distress syndrome (ARDS); the death rate in these patients was as high as 46% [1]. In patients infected with H1N1, the rate of pneumonia was as high as 40%, 25% of patients were admitted into the intensive care unit (ICU), and 36% of those in the ICU developed ARDS [2].

Influenza virus-induced pneumonia is related to an uncontrolled response of the immune system [3–5]. Corticosteroids have been reported to reduce mortality in patients with community-based pneumonia [6]. Patients with life-threatening respiratory failure associated with influenza pneumonia also commonly receive corticosteroids. Animal model studies found that corticosteroid treatment decreased mortality and ameliorated the acute lung injury induced by influenza pneumonia [7, 8]. Steroids might play a role in inhibiting inflammation via mechanisms such as reducing the overproduction of proinflammatory cytokines/chemokines and an excess of activated lymphocytes, which may result in severe lung damage and delayed recovery [9–11]. However, the results of clinical studies of the effect of corticosteroids remain controversial. In some studies, such as Diaz’s study, the use of corticosteroid therapy was not significantly associated with mortality [12], while in others, such as the study of Brun-Buisson, early corticosteroid therapy was found to be potentially harmful in patients with influenza pneumonia [13].

Therefore, based on these controversial findings related to corticosteroid use in adult patients with influenza pneumonia, we conducted a systematic review and meta-analysis of all published trials that have compared mortality between influenza pneumonia patients who received corticosteroid therapy and those who did not. We aimed to identify the roles of corticosteroids and their influence on clinical outcomes in patients with influenza pneumonia.

## Methods

### Search strategies

A literature search was conducted in the PubMed, Embase, Medline, Cochrane Central Register of Controlled Trails (CENTRAL), and Information Sciences Institute (ISI) Web of Science databases using a combination of the following key words: “glucocorticoid” or “corticosteroid” or “steroid” or “cortisone” or “hydrocortisone” “prednisolone” or “methylprednisolone” or “prednisone” or “dexamethasone” or “triamcinolone” and “influenza pneumonia” or “viral pneumonia” without limitations on either the publication type or language. This search was also limited to studies published between 1946 and January 2019. The references listed in each identified article were also screened and manually searched.

### Inclusion and exclusion criteria

Eligible clinical trials were identified based on the following criteria: (1) the subjects enrolled in each study included patients with influenza pneumonia; (2) the patients were divided into an experimental group, in which corticosteroids were applied, and a control group, in which patients were assigned to not receive corticosteroids; and (3) the outcomes included but were not limited to mortality, mechanical ventilation (MV) days, length of stay in the ICU (ICU LOS), and the rate of secondary infection. We excluded studies if they were performed in animals or patients under 18 years old or published as non-controlled studies, reviews, or case reports.

### Study selection

Two independent investigators (YNN and BML) reviewed all titles and abstracts to discard duplicated and non-controlled studies. Then, the full texts of the remaining studies were screened in accordance with previously designed study inclusion criteria to determine eligibility. Disagreements were resolved by a third investigator (ZAL).

### Data extraction

The two researchers independently extracted and recorded desirable information from each enrolled study in a standard form recommended by Cochrane; this information consisted of the authors, the publication year, the study design, the NCT No., population and demographic characteristics (age, gender, etc.), disease conditions (e.g., The Acute Physiologic and Chronic Health Evaluation II (APACHE II), the type of influenza, treatment details (e.g., use of antiviral drugs and the type and initial dose of corticosteroids), scores on the Simplified Acute Physiologic Score II (SAPS II)), the Sequential Organ Failure Assessment (SOFA), and outcome measures (such as mortality, MV days, ICU LOS, and rate of secondary infection). If any of the abovementioned information was not included in a publication, we contacted the corresponding authors by email to obtain the data needed to quantify the measures of association. When the opinions of the two collectors differed, a decision was reached by consensus or consultation with a third investigator.

### Quality assessment

To reduce the risk of bias, all of the studies were independently assessed by two authors (YNN and BML), and the Newcastle-Ottawa Scale was used [14]. Disagreements related to quality assessment were resolved by consensus (Additional file 1).

### Statistical analysis

All statistical analyses performed in the present study were conducted by an independent statistician using Cochrane systematic review software Review Manager (RevMan; Version 5.3.5; The Nordic Cochrane Centre, The Cochrane Collaboration, Copenhagen, 2014). The *Mann-Whitney U test* was performed to test the hypothesis and define statistical significance as a *Z* value and *P* value < 0.05. The results are displayed in Forest plots. Continuous variables are reported as the mean and standard deviation (SD), while dichotomous variables are shown as frequencies and proportions. Statistical heterogeneity was tested by the *χ*^2^ test and qualified as *P* < 0.1 and *I*^2^ > 50%. We also performed a sensitivity analysis to substitute alternative decisions or ranges of values for decisions that were arbitrary or unclear. A random-effects model was applied in the presence of statistical heterogeneity. For continuous data, we calculated the mean difference (MD) and 95% confidence interval (CI), while for dichotomous data, we calculated the risk ratio (RR) and 95% CI. We also performed subgroup analyses according to viral types.

## Results

**Fig. 1:**
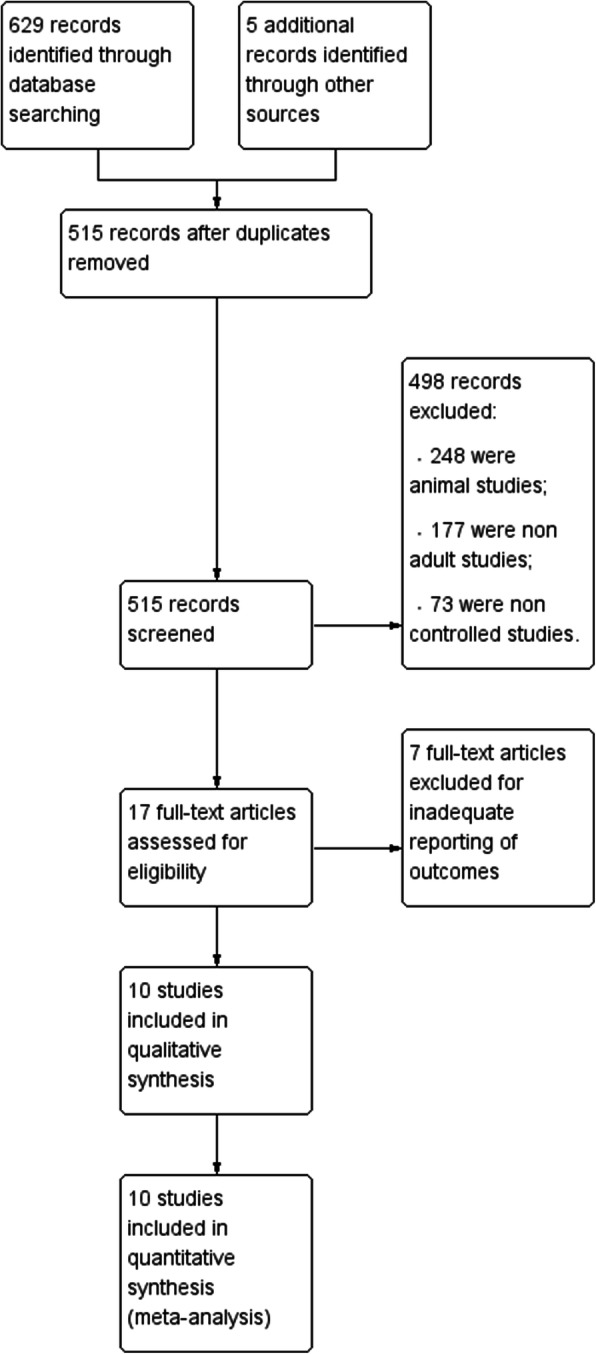
Study flow diagram

A total of 634 records were initially identified. Of these, 629 were extracted from electronic databases, and the remaining 5 were extracted from a review of reference lists (Fig. 1). After screening titles and abstracts, we discarded 617 studies because they were duplicates (*n* = 119), animal experiments (*n* = 248), non-adult patients (*n* = 177), or non-randomized controlled studies (NRCTs, *n* = 73). We searched the full-text articles of the remaining 17 studies, and after we excluded those with inadequate reporting of outcomes (*n* = 7), 10 reports were included in our final analysis.

### Study description

All 10 studies compared outcomes between corticosteroid and non-corticosteroid groups. Mortality was recorded in all 10 studies [12, 13, 15–22], MV days were described in three studies [12, 13, 20], the rate of secondary infection was presented in five studies [12, 13, 15, 19, 20], and the ICU LOS was reported in two studies [13, 20]. Eight studies included patients infected with H1N1, one included patients infected with H7N9 [15], and another included patients with viral A/B/C [20]. The details of each enrolled study are presented in Table 1.

**Table 1:** Characteristics of studies included in the present meta-analysis

Study ID	Study design	RCT no.	Population (corticosteroids/control)	Type of influenza	Type of corticosteroids	Initial dose of corticosteroids (mean ± SD)	Antiviral drug
Brun-Buisson [13]	Retrospective analysis	NR	83/125	H1N1	57.8% hydrocortisone 37.3% methylprednisolone 4.8% prednisone	328 ± 160 (equivalent hydrocortisone)	NR
Cao [15]	Retrospective study	NR	204/84	H7N9	91.7% methylprednisolone 3.9% dexamethasone 2.5% hydrocortisone 2.0% others	81.1 ± 83.2 (equivalent methylprednisolone)	Corticosteroids group: 201/204 Control group: 84/84
Diaz [12]	Prospective observational multicenter study	NR	136/236	H1N1	NR	NR	Corticosteroids group: 136/136 Control group: 236/236
Jung [16]	Multicenter retrospective study	NR	99/120	H1N1	NR	NR	Survivor: 130/141 Death: 68/78
Perez-Padilla [17]	Retrospective study	NR	7/11	H1N1	NR	NR	NR
Lee [18]	Cohort study	NR	264/817	H1N1	NR	NR	151 in all the patients
Li [19]	Case control	NR	1055/1086	H1N1	89.0% methylprednisolone 8.1% dexamethasone 2.0% hydrocortisone 0.9% prednisolone	141.3 ± 142 (equivalent methylprednisolone)	Corticosteroids: 1025/1055 Control group: 1022/1086
Moreno [20]	Secondary analysis of a prospective cohort study	NR	604/1242	Viral A/B/C	95.7% methylprednisolone; 3.8% prednisolone; 0.5% dexamethasone	A median (interquartile range) daily dose equivalent to 80 (60–120) mg of methylprednisolone	NR
Rois [21]	Multicenter prospective study	NR	75/103	H1N1	NR	NR	Survivors: 91/93 Death: 82/85
Viasus [22]	Observational, prospective cohort study	NR	37/160	H1N1	NR	NR	Corticosteroids group: 8/37 Control group: 41/160

*NR* not reported, *SD* standard deviation

A total of 6548 patients were pooled from all the included trials into our final systematic review and meta-analysis. Among these, 2564 patients were treated with corticosteroids, and 3984 were treated with non-corticosteroids. The detailed baseline characteristics of the patients in each enrolled study are shown in Table 2.

**Table 2:** Characteristics of patients included in the present analysis

Study ID	Corticosteroids (*n* = 2564)					Control (*n* = 3984)				
	Age (year)	Male (*n*,%)	BMI	APACHE II	SAPS III	Age (year)	Male (*n*,%)	BMI	APACHE II	SAPS III
Brun-Buisson [13]	49 (34–56)	47 (56.6)	29 (24–33)	NR	51 (44–61)	45 (35–55)	56 (44.8)	27 (23–33)	NR	53 (46–66)
Cao [15]	NR	NR	NR	NR	NR	NR	NR	NR	NR	NR
Diaz [12]	43.1 (12.9)	50.7 (69)	NR	13.25 (6.26)	NR	43.6 (13.6)	57.6 (69)	NR	12.54 (6.7)	NR
Jung [16]	NR	NR	NR	NR	NR	NR	NR	NR	NR	NR
Perez-Padilla [17]	NR	NR	NR	NR	NR	NR	NR	NR	NR	NR
Lee [18]	NR	NR	NR	NR	NR	NR	NR	NR	NR	NR
Li [19]	35.0 (23.8–52.4)	530 (50.2)	NR	NR	NR	33.7 (24.6–48.7)	565 (52)	NR	NR	NR
Moreno [20]	53 (41–62)	357 (59.1)	NR	14 (10–19)	NR	51 (39–61)	739 (59.5)	NR	15 (10–20)	NR
Rois [21]	NR	NR	NR	NR	NR	NR	NR	NR	NR	NR
Viasus [22]	44 (36–53)	15 (40.5)	NR	NR	NR	34 (26–44.5)	13 (41.9)	NR	NR	NR

*APACHEII* Acute Physiologic and Chronic Health Evaluation II, *BMI* body mass index, *NR* not reported, *SAPS III* Simplified Acute Physiologic Score III

### Quality assessment

To ascertain quality, a maximum of nine points was assigned to each study: four for selection, two for comparability, and three for outcomes. A composite score > 6 was regarded as indicative of high quality. Two studies were rated a total score of 9, four studies had a score of 7, and four studies had a score of 6 (Table 3). The funnel plots showed no evidence of publication bias.

**Table 3:** Risk of bias summary

	A. Selection				B. Comparability of cohorts	C. Outcome		
	Represent-activeness of exposed cohort	Selection of non-exposure	Ascertainment of exposure	Outcome not present at start		Assessment of exposure	F/U long enough?	Adequacy of F/U
Brun-Buisson [13]	☆	☆	☆	☆		☆		☆
Cao [15]	☆	☆	☆	☆		☆		☆
Diaz [12]	☆	☆	☆	☆		☆	☆	☆
Jung [16]	☆	☆	☆	☆		☆	☆	☆
Perez-Padilla [17]	☆	☆	☆	☆		☆		☆
Lee [18]	☆	☆	☆	☆		☆	☆	☆
Li [19]	☆	☆	☆	☆		☆	☆	☆
Moreno [20]	☆	☆	☆	☆		☆		☆
Rois [21]	☆	☆	☆	☆	☆☆	☆	☆	☆
Viasus [22]	☆	☆	☆	☆	☆☆	☆	☆	☆

Stars indicate the scores assigned to each study

### Heterogeneity

Significant statistical heterogeneity was found in the analysis of the effect of corticosteroids on mortality (*I*^2^ = 84%, *P* < 0.00001), MV days (*I*^2^ = 53%, *P* = 0.12), and the rate of secondary infection (*I*^2^ = 94%, *P* < 0.00001) in the patients with influenza pneumonia, but not in ICU LOS (*I*^2^ = 38%, *P* = 0.21).

### Mortality

**Fig. 2:**
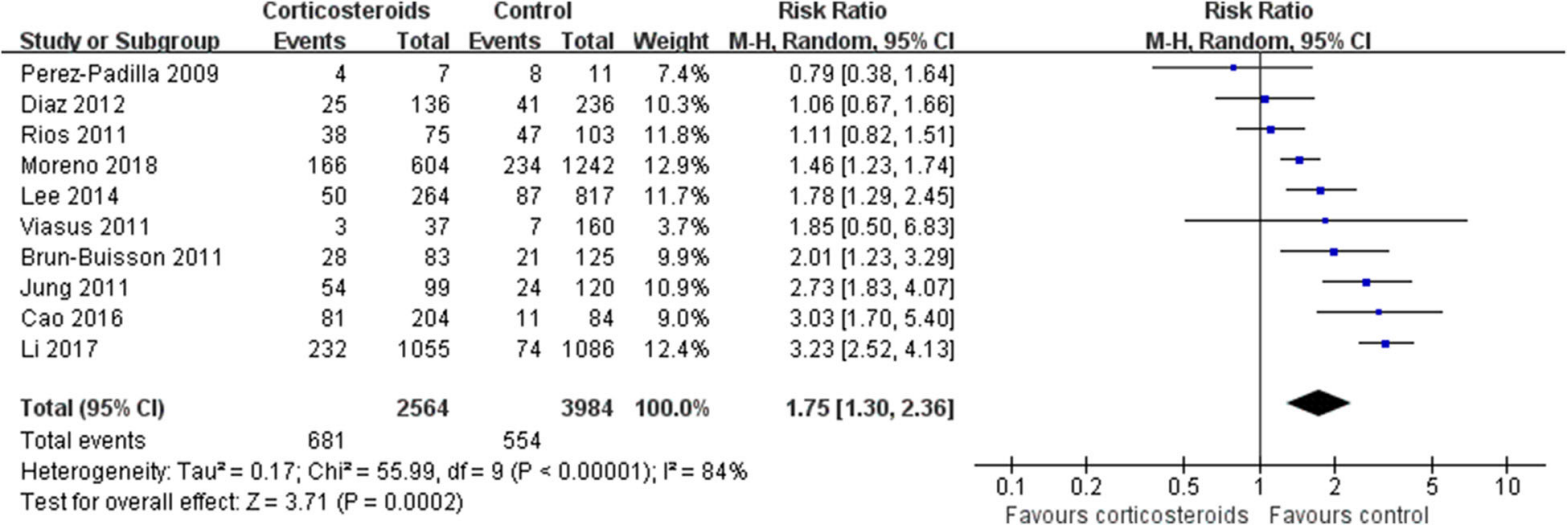
Effect of corticosteroids on mortality. CI, confidence interval; RR, risk ratio

**Fig. 3:**
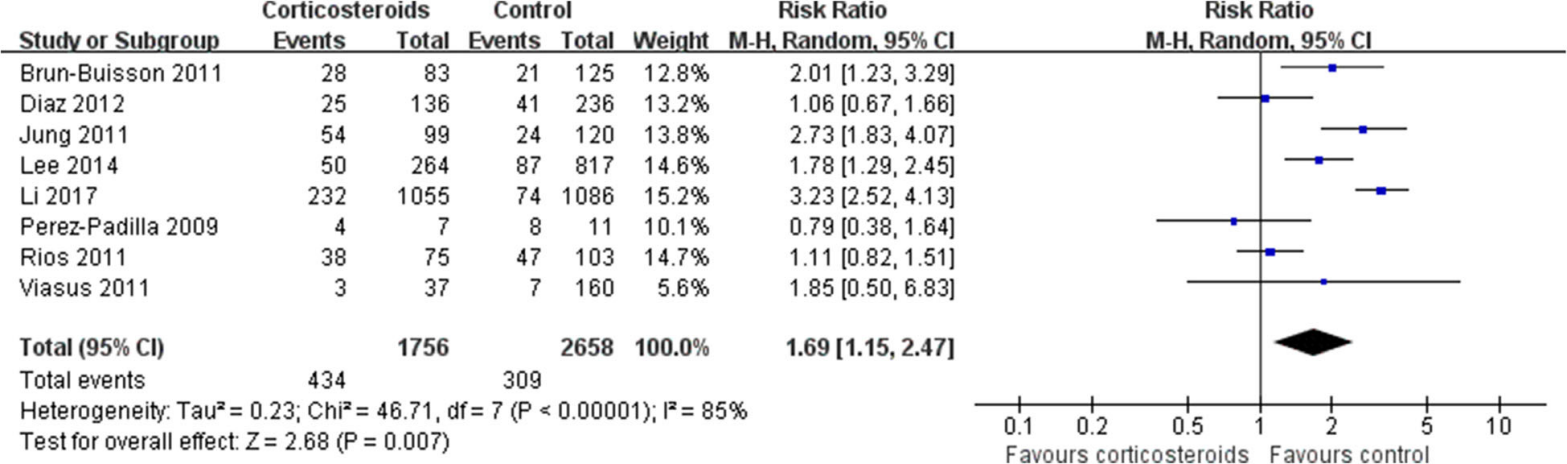
Subgroup analysis of the effect of corticosteroids on mortality in patients with H1N1. Diamonds indicate overall estimates from the meta-analysis; squares indicate point estimates of the result of each study; horizontal lines represent 95% CI. CI, confidence interval; RR, risk ratio

Mortality was higher in patients who received corticosteroids (RR 1.75, 95% CI 1.30 ~ 2.36, *Z* = 3.71, *P* = 0.0002). Similar results were also observed in the subgroup analysis of patients with H1N1 (RR 1.69, 95% CI 1.15 ~ 2.47, *Z* = 2.68, *P* = 0.007) (Figs. 2, and 3) and patients with other viral types (Additional file 2).

### MV days

**Fig. 4:**
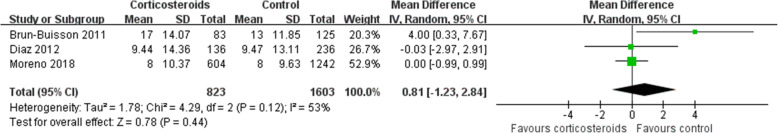
Effect of corticosteroids on MV days. Diamonds indicate overall estimates from the meta-analysis; squares indicate point estimates of the result of each study; horizontal lines represent 95% CI. CI, confidence interval; MV, mechanical ventilation; MD, mean difference

Corticosteroids had no effect on MV days (MD 0.81, 95% CI − 1.23 ~ 2.84, *Z* = 0.78, *P* = 0.44) (Fig. 4). The same result was found in the subgroup analysis (Additional file 2).

### ICU LOS

**Fig. 5:**
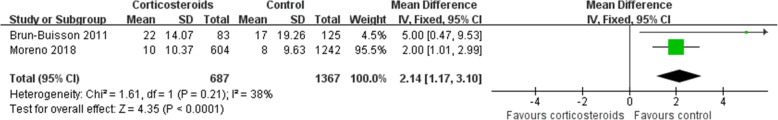
Effect of corticosteroids on ICU LOS. Diamonds indicate overall estimates from the meta-analysis; squares indicate point estimates of the result of each study; horizontal lines represent 95% CI. CI, confidence interval; ICU, intensive care unit; LOS, length of stay; MD, mean difference

ICU LOS was longer in the corticosteroid group (MD 2.14, 95% CI 1.17 ~ 3.10, *Z* = 4.35, *P* < 0.0001) (Fig. 5), and the same result was found in the subgroup analysis (Additional file 2).

### Secondary infection

**Fig. 6:**
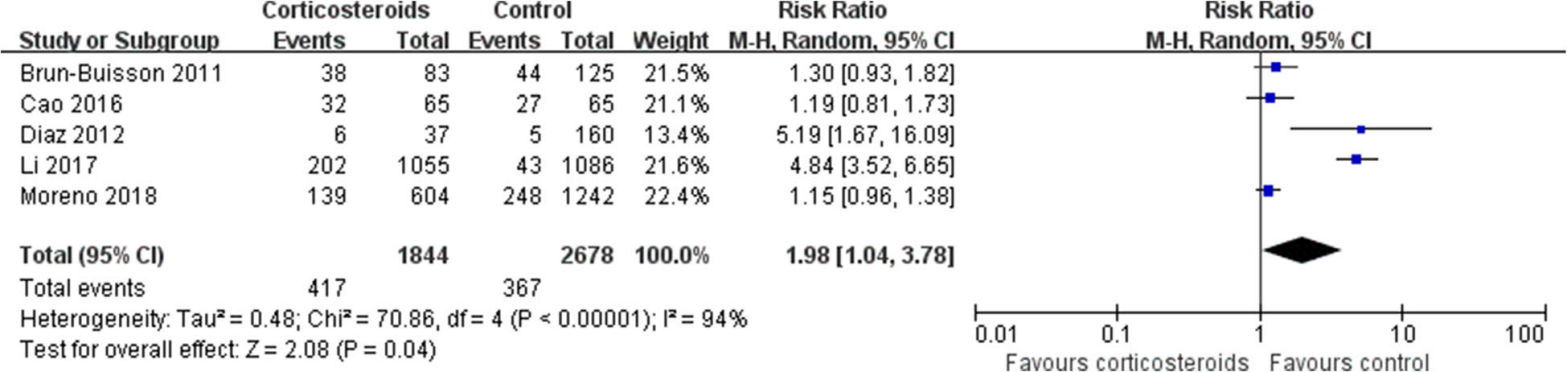
Effect of corticosteroids on the rate of secondary infection. Diamonds indicate overall estimates from the meta-analysis; squares indicate point estimates of the result of each study; horizontal lines represent 95% CI. CI, confidence interval; RR, risk ratio

The rate of secondary infection was higher in patients who received corticosteroids (RR1.98, 95% CI 1.04 ~ 3.78, *Z* = 2.08, *P* = 0.04) than in the control group (Fig. 6), and the same result was found in the subgroup analysis (Additional file 2).

## Discussion

In the present systematic review and meta-analysis, the use of corticosteroids increased mortality, ICU LOS, and the rate of secondary infection in patients with influenza pneumonia but did not influence MV days.

Our analysis demonstrates that corticosteroids not only increase mortality but also prolong ICU LOS. There are several potential mechanisms that could underlie the higher mortality and ICU LOS observed in patients who received corticosteroids. First, corticosteroids reduce systemic inflammation [23]. Once attacked by the virus, the immune system is activated [24]. Corticosteroids inhibit immune reactions by suppressing inflammatory reactions, preventing the migration of inflammatory cells from the circulation to issues by suppressing the synthesis of chemokines and cytokines, and inhibiting immune responses mediated by T cells and B cells [25, 26]. Thus, the alterations in immune reactions caused by corticosteroids might lead to prolonged virus viremia and delay viral clearance, ultimately increasing the risk of mortality [6, 27]. One of our included studies showed that patients who received corticosteroids had lower procalcitonin levels (0.5 vs 0.7 ng/mL, *P* = 0.02) [20], while another showed that the patients who died had a higher rate of immunosuppression (34.7% vs. 15.1%, *P* = 0.02) [13]. Second, our analysis found that patients who received corticosteroids were more likely to develop secondary bacterial pneumonia due to immunosuppression. In addition, longer ICU LOS has also been shown to contribute to secondary infection [28]. Third, due to immune-suppressing effects of corticosteroids, the risk of developing critical illness is increased in corticosteroid-treated patients [29]. One study found that the rate of shock was 8% in the corticosteroid group and 4.4% in the control group [19]. In addition, the invasive MV rate was also increased by corticosteroids, at 38.4% in the corticosteroid group and 4.5% in the control group [19]. Fourth, other corticosteroid-related adverse outcomes, such as cardiovascular events, including fluid retention, premature atherosclerotic disease, and arrhythmias, also increased mortality in patients with influenza pneumonia [30–32]. In the included studies, patients who used more vasopressors had higher mortality [13]. Thus, the above mechanisms may contribute to why patients with influenza pneumonia had higher mortality.

We also performed a subgroup analysis according to viral types. In all types of influenza virus, mortality was higher in those treated with corticosteroids than in controls, although symptoms were more rapidly progressive patients and the risk of ARDS higher in patients infected with H7N9 [1, 2]. Moreover, we included more large sample studies than were included in previous meta-analyses related to influenza [33]. In addition, we focused only on patients with influenza pneumonia and not on those infected with influenza alone or those with influenza who were admitted to the ICU. Influenza pneumonia has been shown to be related to life-threatening respiratory failure and mortality [34]; however, not all patients infected with influenza develop influenza pneumonia. In the present study, we tried to determine whether patients who develop influenza pneumonia benefit from corticosteroids. Nevertheless, we may have omitted patients with influenza pneumonia who were included in trials that studied all influenza patients, and this may have influenced the final results of our analysis.

Studies exploring the effects of corticosteroids on patients with community-based pneumonia have produced positive results [6]. The main reason for these findings is that those infected by bacteria benefit from corticosteroids when given appropriate antibiotic therapy. The early use of antiviral therapy could also reduce mortality. Seven studies reported the use of antiviral therapy. On the one hand, we did not explore the exact role of antiviral therapy in the effects of corticosteroids due to a lack of raw data. On the other hand, we also only included patients who developed influenza pneumonia, which resulted in the included cases being more severe than those included in studies in which patients using antiviral therapy were included.

Moreover, patients who received corticosteroids were more likely to have a superinfection, such as secondary bacterial pneumonia or invasive fungal infection, and exacerbation of underlying conditions, and they also had more prolonged ICU LOS than was found in the no-corticosteroid group [35]. In addition, one study showed that the use of corticosteroids delayed the initiation of neuraminidase inhibitors, with ICU LOS longer in patients who did not receive neuraminidase inhibitors within 5 days of illness [18].

In terms of MV days, corticosteroids did not seem to make a difference. However, only three studies in our analysis reported data on MV days, and the insignificant results might therefore be due to the fact that we had such a small sample size. In other words, a type II error might have occurred because of the limited number of patients.

Other than the aforementioned reasons, the effects of corticosteroids could also be influenced by the following three factors. First, the condition of the respiratory system could be responsible. Corticosteroids can provide benefits to patients with an oxygenation index (OI; partial arterial pressure of oxygen/fraction of inspired oxygen) < 300, but it may also increase the 60-day mortality rate in those with OI > 300 [19]. Second, the time of corticosteroid initiation could be a contributing factor. Compared with no treatment, administration within the first 3 days was more strongly associated with an increased risk of death [13, 36]. Moreover, corticosteroids are beneficial if used early after ARDS onset but otherwise increase mortality. In reality, however, some patients received corticosteroids after ARDS onset, which offset the negative effect of corticosteroids on mortality [37]. Third, the dose of corticosteroids may affect results. High doses of corticosteroids have been associated with greater mortality and a longer duration of viral shedding [15]. In Li’s study, mortality was twice as high in patients who received a high dose of corticosteroids than in those who received a low-moderate dose [19]. The initial dose of corticosteroids varied among our included studies, and some of them did not report related information. Additionally, due to the study design, not all patients in one study received a unified dose of corticosteroids. Moreover, studies have shown that corticosteroids are usually initiated when shock is non-responsive to fluids and vasopressors. Thus, patients who receive corticosteroids tend to have more severe disease, as evidenced by their higher APACHE II scores [36]. It is therefore unclear whether their increased risk of mortality is directly associated with corticosteroid use or due to the severity of disease. None of the studies included in our analysis was a randomized controlled study (RCT). Because the influencing factors could not be controlled, our analysis was highly heterogeneous. This might explain why corticosteroids did not make a difference in some studies.

Despite these findings, the limitations of our study should be addressed. First, the applicability of our study results is limited because none of the studies included in our analysis was an RCT. Second, only two studies reported the dose of corticosteroids and the duration of it use. Third, the baseline characteristics of the patients can influence outcomes and varied among the studies included in our analysis. For example, younger age and fewer underlying diseases might be associated with fewer secondary infections [38]. Finally, the effect of corticosteroids on patients with influenza pneumonia remains controversial. Previous studies that showed a negative effect for corticosteroids may have influenced how the clinicians used corticosteroids in our included studies. Finally, there may have been selection bias because none of the studies included was an RCT.

## Conclusions

Corticosteroids could increase mortality in patients with influenza pneumonia. Randomized controlled studies are needed to further verify this conclusion.

## Additional files

Additional file 1: Assessment of risk of bias and study quality. (DOCX 15 kb)

Additional file 2: Subgroup analysis according to virus type. (DOCX 88 kb)

## Abbreviations

APACHE II: Acute Physiologic and Chronic Health Evaluation II
CENTRAL: Cochrane Central Register of Controlled Trials
CI: Confidence interval
ICU: Intensive care unit
ISI: Information Sciences Institute
LOS: Length of stay
MD: Mean difference
MV: Mechanical ventilation
OI: Oxygen index
RR: Risk ratio
SAPS III: Simplified Acute Physiologic Score III
SD: Standard deviation
SOFA: Sequential Organ Failure Assessment
