# Correction to: The effect of corticosteroids on the mortality of patients with influenza pneumonia: a systematic review and meta-analysis

**DOI:** 10.1186/s13054-020-02996-2

**Published:** 2020-06-23

**Authors:** Yue-Nan Ni, Guo Chen, Jiankui Sun, Bin-Miao Liang, Zong-An Liang

**Affiliations:** 1 Department of Respiratory and Critical Care Medicine, West China School of Medicine and West China Hospital, Sichuan University, No.37 Guoxue Alley, Chengdu, 610041 Sichuan China; 2 Department of Geriatrics, Sichuan Academy of Medical Sciences & Sichuan Provincial People’s Hospital, Chengdu, Sichuan China; 3 State Key Laboratory of Oral Diseases, West China School of Stomatology, Sichuan University, No.14, Section 3 Renmin Nanlu, Chengdu, 610041 Sichuan China

**Correction to: Critical Care (2019) 23:99**

**https://doi.org/10.1186/s13054-019-2395-8**

After publication of our article [1], we were made aware of some errors in our figures and tables. There have been no changes to the interpretation of the results, conclusions and applications of our article.

In Fig. 2 and Fig. 3, for Perez-Padilla 2009, the events/total in the corticosteroids and control groups should be 3/5 and 4/13, respectively, instead of 4/7 and 8/11 in the original article. For Viasus 2011, the events/total in the corticosteroids and control groups should be 3/37 and 4/160, respectively, instead of 3/37 and 7/160 in the original article. Thus, in our final systematic review and meta-analysis, 2,562 patients were treated with corticosteroids and 3,986 with non-corticosteroids. The statistical heterogeneity in the analysis of the effect of corticosteroids on mortality should be (I^2^=83%, P<0.00001), instead of (I^2^=84%, P<0.00001) in the original article. And, the results of the analysis about mortality should be (RR 1.91, 95% CI 1.42~2.55, Z=4.33, P<0.0001), instead of (RR 1.75, 95% CI 1.30~2.36, Z=3.71, P=0.0002). Similarly, the results of the subgroup mortality in H1N1 patients should be (RR 1.92, 95% CI 1.23~3.02, Z=2.85, P=0.004), rather than (RR 1.69, 95% CI 1.15~2.47, Z=2.68, P=0.007).

Otherwise, Lee 2014 should be Lee 2015.

**Fig. 2:**
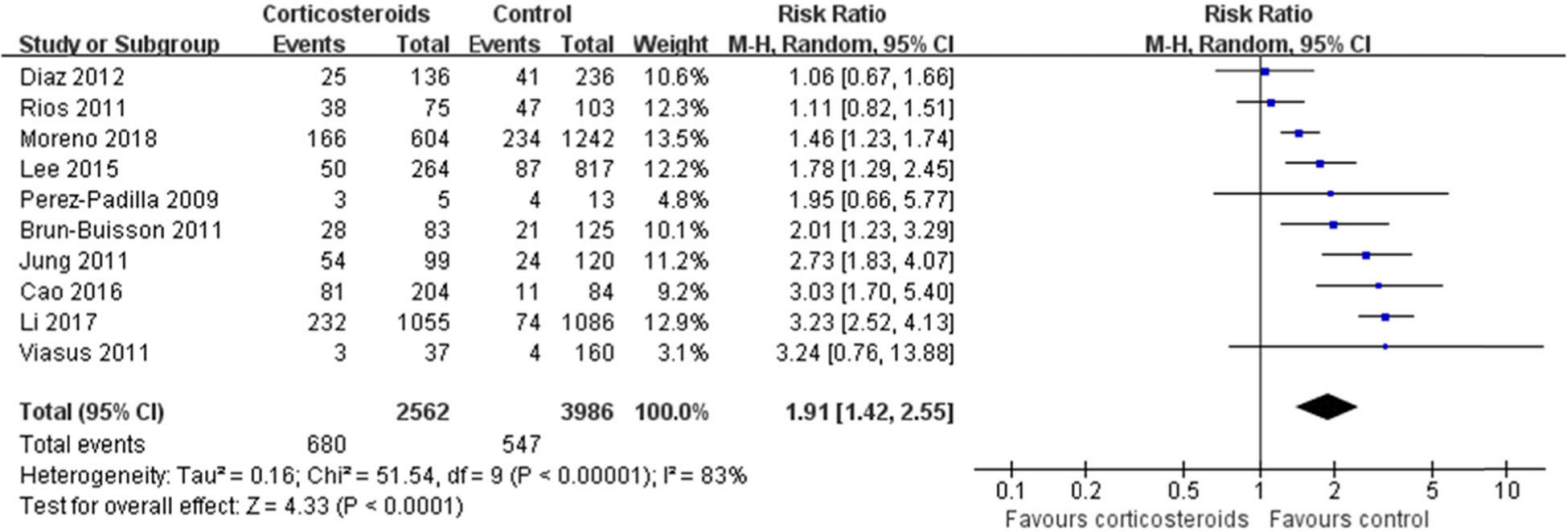
Effect of corticosteroids on mortality

**Fig. 3:**
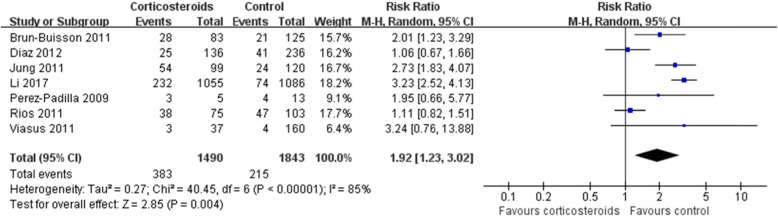
Subgroup analysis regarding the effect of corticosteroids on mortality in patients with H1N1

In Fig. 5, the SD for the corticosteroids and control groups in Brun-Buisson’s study should be 19.26 and 14.07, respectively, instead of 14.07 and 19.26 in the original article. Thus, the statistical heterogeneity of the analysis on ICU LOS should be (I^2^=30%, P=0.23), instead of (I^2^=38%, P=0.21). And the result of this analysis should be (MD 2.12, 95% CI 1.15~3.09, Z=4.30, P<0.0001), rather than (MD 2.14, 95% CI 1.17~3.10, Z=4.35, P<0.0001) in the original article.

**Fig. 5:**
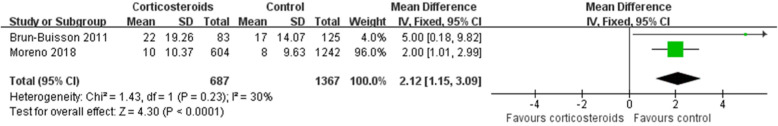
Effect of corticosteroids on ICU LOS

In Fig. 6, the study ID “Dias 2012” should be “Viasus 2011”.

**Fig. 6:**
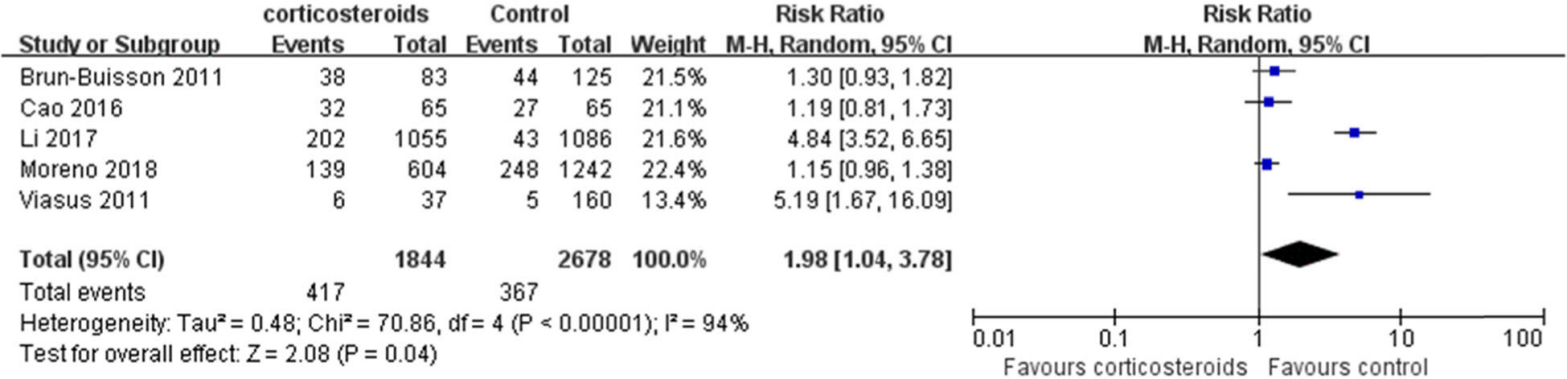
Effect of corticosteroids on the rate of secondary infection

In table 1, the antiviral drug in Lee’s study should be NR.

In table 2, for Brun-Buisson’s study, the Male (n, %) in the corticosteroids group should be 36(43.4), and 69(55.2) in the control group. For Moreno’s study, the APACHE II in the corticosteroids group should be 15(10-20) and 14(10-19) in the control group.

And, the age and male in the control group of Viasus’s study should be NR.
