# Supplementary material for: The effect of corticosteroids on mortality of patients with influenza pneumonia: a systematic review and meta-analysis
Source: Crit Care. 2019 Mar 27;23:99. doi: 10.1186/s13054-019-2395-8 (PMC6437920; doi:10.1186/s13054-019-2395-8)
Supplement: Supplementary file 1 — Assessment of risk of bias and study quality. (DOCX 15 kb) [file 13054_2019_2395_MOESM1_ESM.docx]

**Additional file 1 Assessment of risk of bias and study quality**

Note: A study can be awarded a maximum of one star for each numbered item within the Selection and Outcome categories. A maximum of two stars can be given for Comparability The star could be awarded when the item marked by * was achieved.

**A. Selection**

**1) Representativeness of the exposed cohort**

a) Truly representative of the average cohort of patients with influenza pneumonia *

b) Somewhat representative of the average cohort of patients with influenza pneumonia *

c) Selected group of users e.g. nurses, volunteers

d) No description of the derivation of the cohort

**2) Selection of the non-exposed cohort**

a) Drawn from the same community as the exposed cohort *

b) Drawn from a different source

c) No description of the derivation of the non-exposed cohort

**3) Ascertainment of exposure**

a) Secure record (e.g. medical records) *

b) Structured interview *

c) Written self report

d) No description

**4) Demonstration that outcome of interest was not present at start of study**

a) Yes *

b) No

**B. Comparability**

**1) Comparability of cohorts on the basis of the design or analysis**

a) Study controls for severity of illness *

b) Study controls for ALL of the following: age, co-morbidity, and clinical risk of influenza pneumonia *

**C. Outcome**

**1) Assessment of outcome**

a) Independent blind assessment *

b) Record linkage *

c) Self report

d) No description

**2) Was follow-up long enough for outcomes to occur**

a) Yes (hospital stay considered adequate follow up period for outcome of interest) *

b) No

**3) Adequacy of follow up of cohorts**

a) Complete follow up - all subjects accounted for *

b) Subjects lost to follow up unlikely to introduce bias; small number lost to follow-up (> 90 % follow up), or description provided of those lost) *

c) Follow up rate < 90% and no description of those lost

d) No statement
