# Supplementary material for: The effect of corticosteroids on mortality of patients with influenza pneumonia: a systematic review and meta-analysis
Source: Crit Care. 2019 Mar 27;23:99. doi: 10.1186/s13054-019-2395-8 (PMC6437920; doi:10.1186/s13054-019-2395-8)
Supplement: Supplementary file 2 — Subgroup analysis according to virus type. (DOCX 88 kb) [file 13054_2019_2395_MOESM2_ESM.docx]

**Figure S1** Subgroup analysis of mortality

**
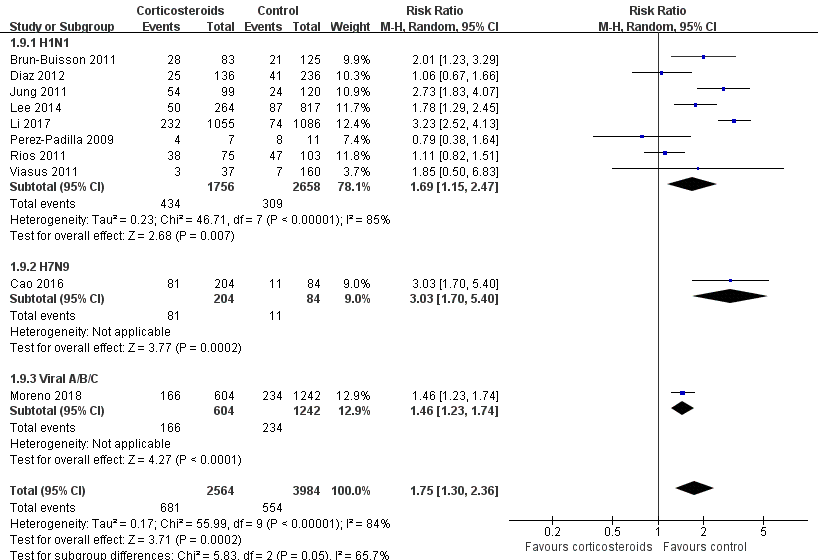
**

Diamonds indicate overall estimates from the meta-analysis; squares indicate point estimates of the result of each study; horizontal lines represent 95% CI.

CI, confidence interval; RR, risk ratio

**Figure S2** Subgroup analysis of MV days


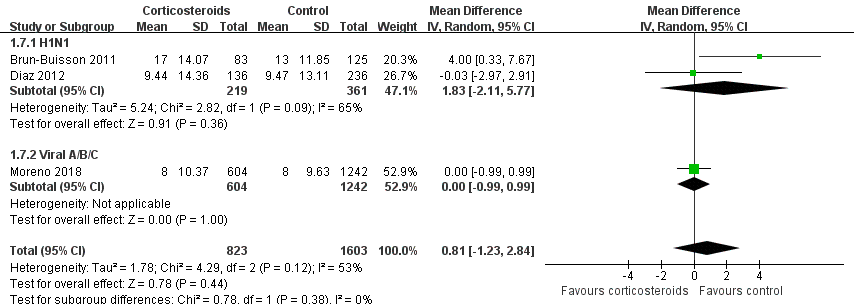


Diamonds indicate overall estimates from the meta-analysis; squares indicate point estimates of the result of each study; horizontal lines represent 95% CI.

CI, confidence interval; MV, mechanical ventilation; MD, mean difference

**Figure S3** Subgroup analysis of ICU LOS


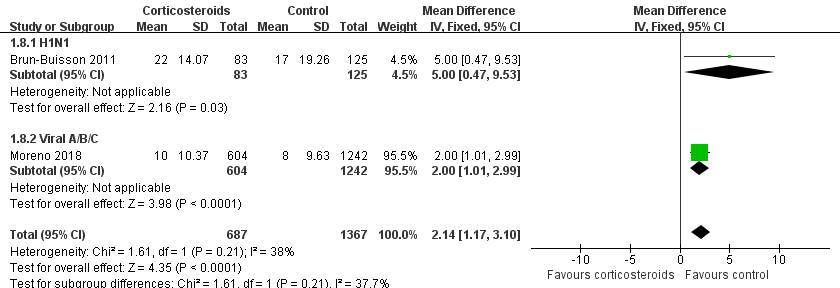


Diamonds indicate overall estimates from the meta-analysis; squares indicate point estimates of the result of each study; horizontal lines represent 95% CI.

CI, confidence interval; ICU, intensive care unit; LOS, length of stay; MD, mean difference

**Figure S4** Subgroup analysis of rate of secondary infection


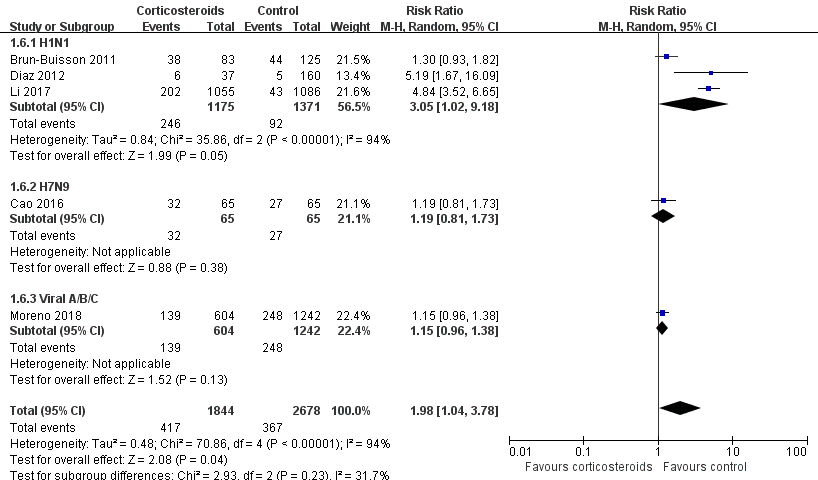


Diamonds indicate overall estimates from the meta-analysis; squares indicate point estimates of the result of each study; horizontal lines represent 95% CI.

CI, confidence interval; RR, risk ratio
